# Supplementary material for: N-Rich, Polyphenolic Porous Organic Polymer and Its In Vitro Anticancer Activity on Colorectal Cancer
Source: Molecules. 2022 Oct 28;27(21):7326. doi: 10.3390/molecules27217326 (PMC9657835; doi:10.3390/molecules27217326)
Supplement: Supplementary file 1 [file molecules-27-07326-s001.zip › molecules-1957862-supplementary.pdf]

## Supporting Information

# N-rich, polyphenolic porous organic polymer and its *invitro* anticancer activity on colorectal cancer

*Sabuj Kanti Das,‡ Snehasis Mishra‡, Krishna Das Saha, Debraj Chandra, Michikazu Hara, Amany A. Mostafa and Asim Bhaumik**

**Corresponding author. E-mail: msab@iacs.res.in*

‡*These authors contributed equally.*

**Table of Contents**

**Figure S1** FTIR spectra of TrzTFPPOP after DI water, 2 (M) $H_2SO_4$ and 2 (M) NaOH treatments for 7 days.

**Figure S2** Wide angle PXRD pattern of TrzTFPPOP.

**Figure S3** MTT data of (0-10 μg/ml) TrzTFPPOP against HCT 116 cell line. The $IC_{30}$, $IC_{50}$ and $IC_{70}$ values are in the inset.

**Figure S4** MTT data of (0-20 μg/ml) TrzTFPPOP against HEK293 cell line

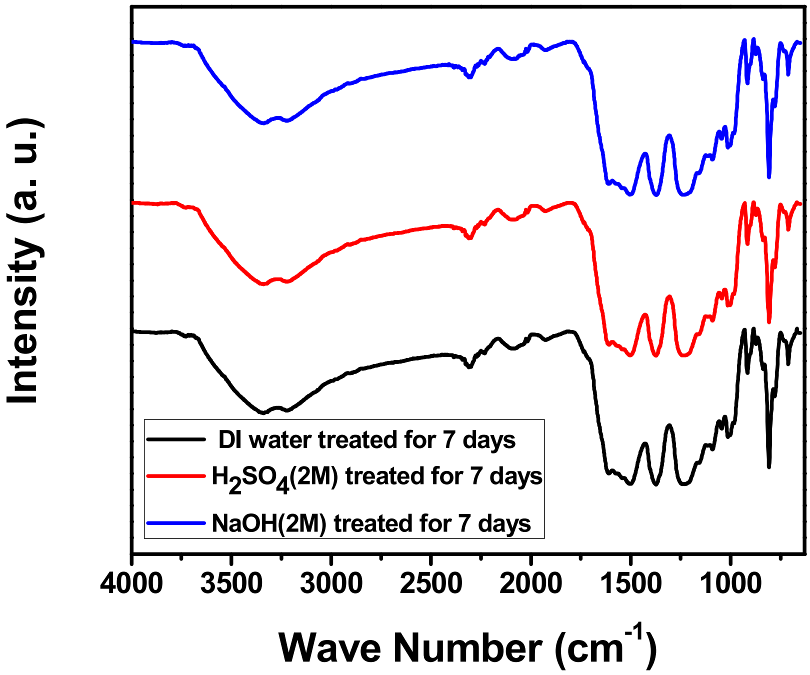


**Figure S1:** FTIR spectra of TrzTFPPOP after DI water, 2 (M) $H_2SO_4$ and 2 (M)NaOH treatment for 7 days.

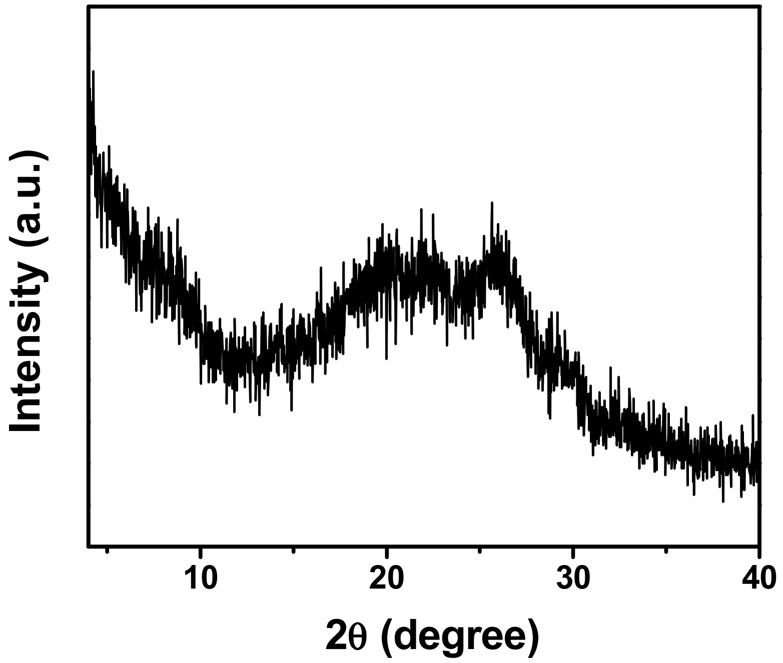


**Figure S2:** Wide angle PXRD pattern of TrzTFPPOP.

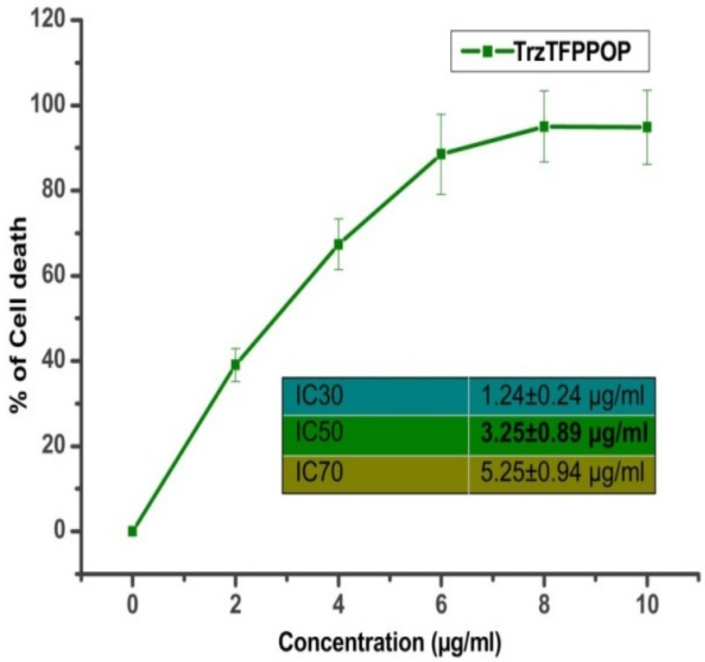


**Figure S3**: Percentage of cell death after treatment of (0-10 μg/ml) TrzTFPPOP. The $IC_{30}$, $IC_{50}$ and $IC_{70}$ values are in the inset.

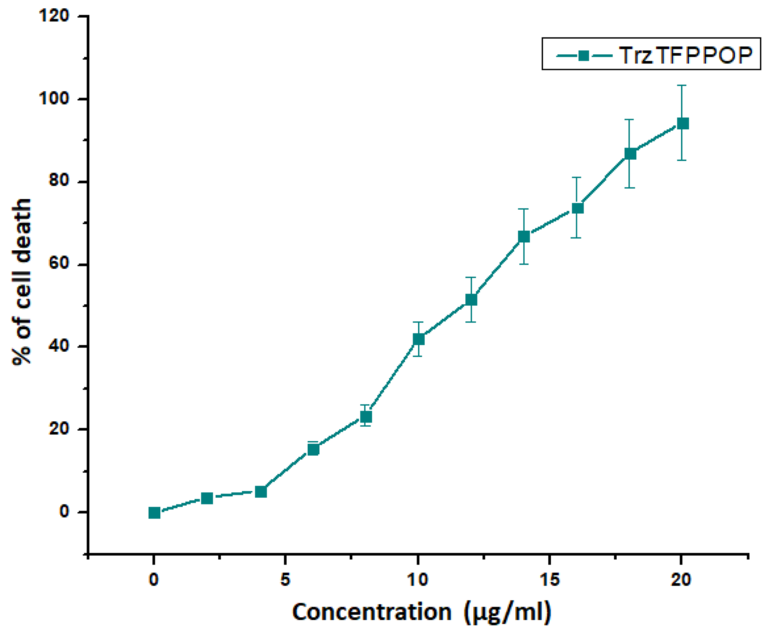


**Figure S4**: Percentage of cell death after treatment of (0-10 µg/ml) TrzTFPPOP in HEK293 cell line.
